# Encoded Expansion: An Efficient Algorithm to Discover Identical String Motifs

**DOI:** 10.1371/journal.pone.0095148

**Published:** 2014-05-28

**Authors:** Aqil M. Azmi, Abdulrakeeb Al-Ssulami

**Affiliations:** Department of Computer Science, College of Computer & Information Sciences, King Saud University, Riyadh, Saudi Arabia; The University of Hong Kong, Hong Kong

## Abstract

A major task in computational biology is the discovery of short recurring string patterns known as motifs. Most of the schemes to discover motifs are either stochastic or combinatorial in nature. Stochastic approaches do not guarantee finding the correct motifs, while the combinatorial schemes tend to have an exponential time complexity with respect to motif length. To alleviate the cost, the combinatorial approach exploits dynamic data structures such as trees or graphs. Recently (Karci (2009) Efficient automatic exact motif discovery algorithms for biological sequences, Expert Systems with Applications 36:7952–7963) devised a deterministic algorithm that finds all the identical copies of string motifs of all sizes 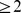 in theoretical time complexity of 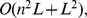 and a space complexity of 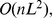 where 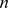 is the length of the input sequence and 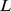 is the length of the longest possible string motif. In this paper, we present a significant improvement on Karci's original algorithm. The algorithm that we propose reports all identical string motifs of sizes 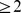 that occur at least 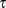 times. Our algorithm starts with string motifs of size 2, and at each iteration it expands the candidate string motifs by one symbol throwing out those that occur less than 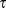 times in the entire input sequence. We use a simple array and data encoding to achieve theoretical worst-case time complexity of 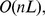 and a space complexity of 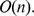 Encoding of the substrings can speed up the process of comparison between string motifs. Experimental results on random and real biological sequences confirm that our algorithm has indeed a linear time complexity and it is more scalable in terms of sequence length than the existing algorithms.

## Introduction

There has been a considerable interest in motif discovery from both computer scientists and computational biologists. Motifs or binding sites are significant for understanding the mechanism behind regulating gene expressions. From a biological sequence analysis perspective, the significant pattern is the substring that is either over-represented or under-represented in a biological sequence. Therefore, the main problem is to identify the most or the rarest recurring patterns. Some of the methods depend on comparing the biological sequence with the background sequences to discover exceptional motifs. Those methods require generating specific length background sequences randomly. The problem of deciding whether the generated sequences respect the motif constraints, the number of motif occurrences, the length of motif, is NP-Complete [1]; essentially meaning that it is computationally hard for all practical purposes. To solve this problem we require an exponential time complexity, though space complexity may not be exponential.

Over the years, many algorithms were developed to discover and report motifs; and most of these algorithms were either stochastic or combinatorial in nature [2]. The stochastic algorithms such as Expectation Maximization (EM) [3], take a set of input sequences, the motif length, and an initial guess for the motif. This guess is generated either randomly or supplied by the user. The algorithm returns a probabilistic model of the consensus pattern, or the motif. EM assumes that there is a single motif occurring in each input sequence. It is possible that EM fails to return the correct motif, that is if we were unfortunate in picking a good starting point. Subsequently newer algorithms came into existence that extended EM, e.g. Gibbs Sampling [4], [5], [6], [7], [8], [9], and Multiple EM for Motif Elicitation (MEME) algorithm [10], [11], [12], [13], [14], [15]. On the other hand, the combinatorial approaches are very expensive because they exhaustively generate and search for each possible permutation of a given length making them impractical for motif sizes over 10 [16]. Some of the algorithms that fall into this category includes: Weeder [17], MotifEnumerator [18], Seeder [19], the algorithm by Marschall and Rahmann [20], VINE [21], PairMotif [22], and PairMotif+ [23]. Few of these algorithms resort to smart pruning to reduce the search space [17], [19], [23].

Most of the above algorithms rely on dynamic data structure to process the data. When it comes to the insertion and deletion operations, the dynamic data structures are very efficient. However, traversing dynamic data structures is less efficient than doing so on the static data structures as the data might be scattered all around [24], [25]. Static data structures, e.g. arrays, are preferred if the algorithm was properly crafted. Our goal is to devise an efficient algorithm that discovers all the identical string motifs and does not rely on dynamic data structure thus saving on the memory requirements. The algorithm must be highly efficient and fast enough to compete with others in the same class. Recently, Karci [26] proposed a deterministic algorithm that reports all identical string motifs of all possible sizes 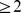 if they occur at least twice in the entire input sequence. The original algorithm by Karci was highly inefficient, both in terms of time and space. Our implementation of the algorithm in [26] has significant improvements in terms of time and space complexities along with the ability of the user to set a threshold 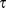 for the minimum number of motifs. Karci's algorithm lacks this feature always assuming a constant 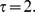


Since this work is inspired by Karci, it is reasonable for us to cover his algorithm in more detail. The author introduced the word CanMotifs (candidate motifs), a term we borrow and use in this paper to refer to candidate string motifs. A substring of any size is considered a CanMotif and if we can find at least one more copy of the same identical substring then it is called motif. The basic idea in [26] is to keep expanding CanMotifs one nucleotide at a time, starting from the pair bases that occur in the input sequence, and then continue with an exhaustive search. To generate 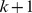
–lets CanMotifs each of the 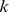
–lets CanMotifs is augmented twice, once using the nucleotide to its immediate left and the other time using the nucleotide to its immediate right. This is followed by the process of eliminating duplicate entries. To better illustrate Karci's algorithm, consider for example the sequence: AACTGCTACTT. It starts by generating the pair bases: {(AA, 1), (AC, 2), (CT, 3), (TG, 4), …, (TT, 10)}, where the tuple stands for (the substring, its starting position). Now to find out all the 2–lets string motifs, the algorithm goes through the full list looking for identical CanMotifs but with different starting positions. Next, it generates 3–lets CanMotifs. These are generated by augmenting each of the 2–lets CanMotifs twice, e.g. AC→AAC and ACT (using left and right nucleotide respectively), similarly CT→ACT and CTG. The 3–lets CanMotifs are: {(AAC, 1), (AAC, 1), (ACT, 2), (ACT, 2), (CTG, 3), …}. This expansion results in duplicate entries which have to be eliminated. It is followed by a search process to find all the 3–lets string motifs. The procedure continues till no more string motifs are discovered. This algorithm has a time complexity of 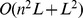 and a space complexity of 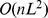 where 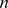 is the size of the input sequence and 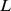 is the length of the longest possible string motif. Clearly, the algorithm reports all the identical string motifs but there are plenty of unnecessary steps.

We named our algorithm Encoded Expansion. The idea of expansion comes from the original algorithm in [26] although it is a much improved approach, and the encoding is from our quest of finding a more efficient way to compare between CanMotifs. With these improvements, we devise a very efficient algorithm to discover all identical string motifs. Theoretically, our algorithm has a worst-case time complexity of 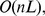 and a space complexity of 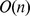 where 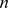 is the size of the input sequence and 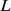 is the length of the longest possible string motif that occurs at least 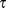 times. We tested out the algorithm on random sequences as well as some real biological sequences of different sizes and it shows our scheme to be linear in time. In the subsequent discussion, the term motif will always refer to string motif. Similarly, CanMotif will refer to candidate string motif.

## Proposed Algorithm

Let 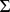 denote the set of finite symbols (alphabet), we define 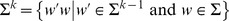 where 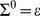 (empty string) to be a set composed of elements each of length 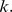 The 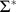 denotes the set of all strings formed using the symbols in 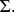 In our case the alphabet 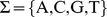 contains the four bases for the DNA biological sequence, so that any DNA sequence belongs to the language 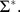 In this work we assume that all the indices start from 0. Let *S* be a string of length *n*, 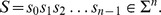 Substrings of *S* which start at position *p* and are of length *k* are denoted 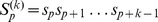 where 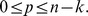


Our objective is: given the sequence *S*, find all the identical string motifs of lengths up to 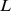 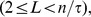 where all motifs of length 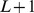 appears less than 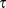 times in 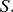 A motif of length *L* is a substring that is repeated at least 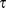 times in different positions in *S*.

Our algorithm proceeds as follows. We start by generating the pair bases (2–lets CanMotifs) and their starting positions. For example, if *S* = TATAC and 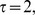 then the list is {(TA, 0), (AT, 1), (TA, 2), (AC, 3)} where the tuple stands for (CanMotif, starting position). The algorithm encodes the 2–lets CanMotifs using the codes in Table 1, resulting in {(12, 0), (3, 1), (12, 2), (1, 3)}. Next the encoded sequence is sorted. Sorting is achieved by going over the list sixteen times. For our example, the list after the sorting will be {(1, 3), (3, 1), (12, 0), (12, 2)}. Then we delete all the entries where the encoded CanMotif occured less than *τ* times resulting in {(12, 0), (12, 2)}. What remains is a list of all 2–lets motifs. The algorithm will proceed to the next stage only if it was successful in discovering motif(s) at the current stage.

**Table 1:** Encoding of pair bases.

AA	AC	AG	AT	CA	CC	CG	CT	GA	GC	GG	GT	TA	TC	TG	TT
0	1	2	3	4	5	6	7	8	9	10	11	12	13	14	15

This is the full list of all 2–lets candidate motifs (CanMotifs).

We start the next stage by generating an encoded list of all 3–lets CanMotifs. The list is generated by augmenting every occurrence of 2–lets motifs (out of the previous stage) with one nucleotide to its right in the input sequence *S*. Going back to our previous example we have only one 2–lets motif, TA. The generated 3–lets CanMotifs are TAT and TAC which will be encoded (more on that later). Once the encoded list is sorted we delete all CanMotifs that occur less than the threshold 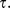 The remainder is a list of all 3–lets motifs. The process continues discovering larger motifs and stops when there are no more left, or if they occur less than 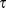 times. There are three aspects of the algorithm that will be covered in depth including the generation of the CanMotifs, the encoding scheme; and finally sorting and discovery of the motifs. For our ensuing discussion we would like to denote 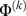 for the set of *k*–lets CanMotifs.

### Generating CanMotifs

**Figure 1:**
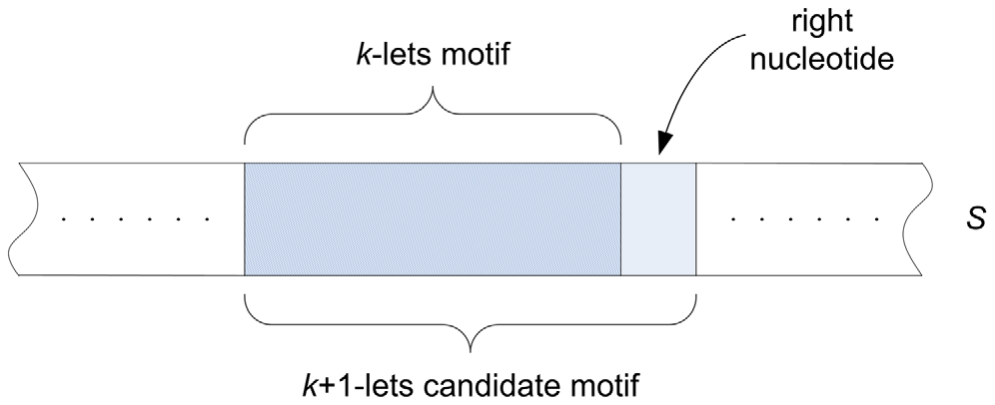
Each occurrence of *k*–lets motif is augmented with right nucleotide to form the 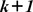
–lets CanMotif.

In our algorithm we use *k*–lets motifs to generate the list of 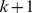
–lets CanMotifs. Since there are only sixteen 2–lets CanMotifs, we generate them all and this scheme is applied for 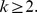 This is done by augmenting each occurrence of the *k*–lets motif with one right nucleotide. Figure 1 illustrates our scheme.

There are two issues that need to be addressed: (1) why did we not use *k*–lets CanMotifs to generate the list of 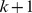
–lets CanMotifs; and (2) what difference (if any) does it make if we augment the *k*–lets motifs with one left nucleotide instead of one right nucleotide in our algorithm.

**Theorem 1**. It is redundant to use *k*–lets CanMotifs over *k*–lets motifs to generate the 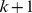
–lets CanMotifs.

**Proof 1.** It suffices to show that a 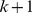
–lets motif cannot be derived from a non-motif substring of length *k*. Let 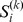 be a substring of length *k* of the string *S*. Assume that 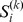 is not a motif. Therefore there are no substrings 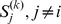 in *S* which equals 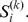 Now, in the best case, both substrings share at most 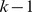 symbols, otherwise they will be equal and 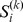 is a motif. Augmenting 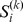 and 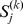 with a single symbol each gives us at best a *k*–lets motif which is not what we are looking for.

A consequence of the above is a faster algorithm since the set of *k*–lets motifs is much smaller than the set of *k*–lets CanMotifs. For example, consider the sequence, AACTGCTACTT. There are 10 2–lets CanMotifs: AA, AC, CT, TG, GC, CT, TA, AC, CT and TT, but only two 2–lets motifs: AC and CT. These 2–lets motifs will be augmented by one right nucleotide to form the set of 3–lets CanMotifs.
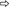


**Theorem 2.** Augmenting the *k*–lets motifs in either direction (left or right) to generate the list of 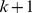
–lets CanMotifs will yield the same set of 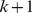
–lets motifs.

**Proof 2.** Note that a CanMotif may or may not yield a motif, and that two different CanMotifs cannot yield the same motif. It does not matter if left and right augmentations result in a different set of CanMotifs, the important thing is that they both result in identical motifs. Suppose that we have two identical substrings of length 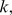 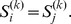 Let 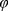 represent this *k*–lets motif. Note that 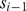 is the left nucleotide of 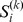 and 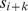 is its right nucleotide, for 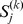 is 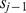 and 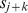 respectively. Augmenting the *k*–lets motif 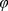 with the left nucleotide results in the following 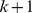
–lets CanMotifs: 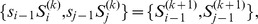 while augmenting 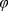 with the right nucleotide results in the 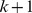
–lets CanMotifs: 
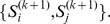
 Depending on whether the left and the right nucleotides are the same or not, we have four different cases:

Case 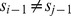 and 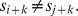 Here neither augmentation of the *k*–lets motif 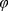 will yield a 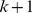
–lets motif.

**Figure 2:**
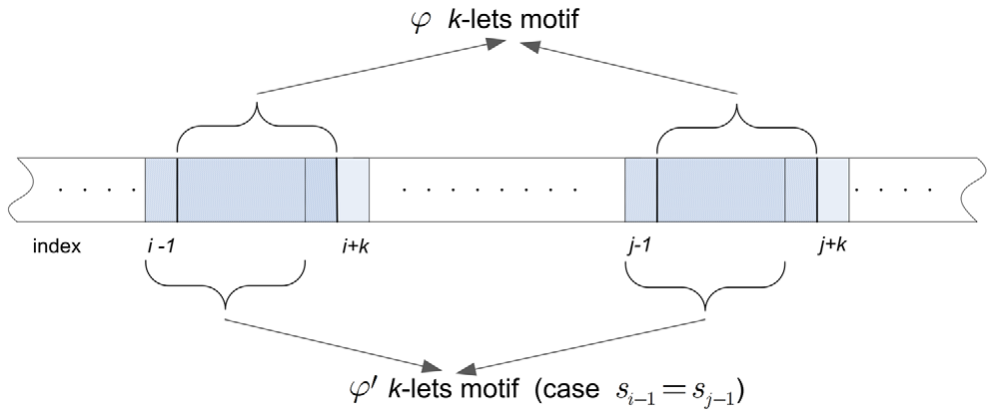
Left augmentation of 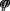 and the right augmentation of 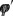 yield the same 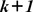
–lets motif.

Case 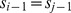 and 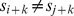 (Figure 2). Since 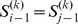 we have another 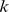
–lets motif, 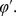 To shorten the argument we will assume that 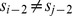 and 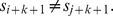 Now the left augmentation of the *k*–lets motif 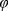 will be the same as the right augmentation of the *k*–lets motif, 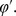 In other words both will yield the same 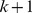
–lets motif. Certainly, the right augmentation of 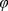 and the left augmentation of 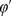 will yield a 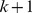
–lets CanMotif, one which will not result in a motif.

Case 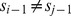 and 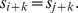 Argument similar to above.

Case 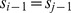 and 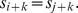 This is a combination of the above two cases.
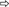


Therefore, it is possible to use either augmentation. Using the left augmentation results in a different set of CanMotifs than using the right augmentation, nonetheless both will yield the same set of motifs.

### CanMotifs encoding scheme

The basic idea is to map a *k*–lets CanMotif 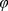 to a single integer. Thus, a comparison of substrings will be just a comparison between integers. Comparing two substrings of length *k* for exact match requires (in the worst case) *k* comparisons versus a single compare operation between integers. We want a one-one mapping between the *k*–lets CanMotifs and 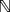 (the set of non-negative integers),
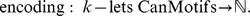



That is CanMotifs 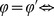 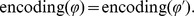 This condition is necessary or else we will have erroneous results. Note that there are sixteen 2–lets CanMotifs, sixty-four 3–lets CanMotifs and in general the maximum number of *k*–lets CanMotifs is 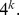 Table 2 shows a simple encoding scheme which guarantees that no two different CanMotifs have the same code. For example, for 3–lets the range is between 16 to 79, so encoding(AAA) = 16, encoding(AAC) = 17 and encoding(TTT) = 79. And for 4–lets, encoding(AAAA) = 80 and encoding(TTTT) = 335. We can even write a simple inverse function to map back the encoding to the CanMotif itself.

**Table 2:** Encoding of CanMotifs with different lengths (*k*).

*k*	Max possible CanMotifs	First value	Last value
2	16	0	15
3	64	16	79
4	256	80	335
5	1024	336	1359
6	4096	1360	5455
7	16384	5456	21839
8	65536	21840	87375
9	262144	87376	349519
10	1048576	349520	1398095

However, if we insist on a unique encoding for each different CanMotif of every size then we will soon run out of range, which is imposed by the 32-bit integer. A simple calculation shows that at 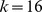 we will have an integer overflow. We want our algorithm to handle motifs of any size as a result the above encoding is inappropriate. Our proposed solution is to re-encode (re-number) the discovered 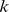
–lets motifs so it always starts from 0. This way we eliminate the risk of integer overflow. More on that later.

**Algorithm 1.** Algorithm to generate 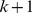
–lets encoded CanMotifs. This algorithm is for cases when 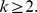


**Input.** Sequence 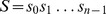 and list of encoded *k*–lets motifs of the form 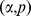 where the tuple stands for (encoded *k*–lets motif, its starting position)

**Output.** List of encoded 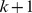
–lets CanMotifs of the form 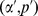 which stands for (encoded 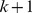
–lets CanMotif, starting position)

**Begin.**


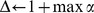
//value of largest encoded motif

**Loop** over all encoded *k*–lets motifs

**{**

  if 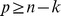 then continue //reached boundary


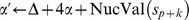



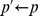


**}**

**End.**

The data is encoded using the tuple (encoded CanMotif, starting position). The starting position helps in recovering the CanMotif and in fetching the right nucleotide which the motif has to be augmented with.

We use Table 1 for encoding 2–lets CanMotifs. For larger CanMotifs, we use Algorithm 1 to generate and encode 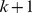
–lets CanMotifs out of encoded *k*–lets motifs.

We want to avoid a situation where the encoding of the generated CanMotif is the same as that of the encoding of one of the input motifs. The variable Δ is used to take care of this problem. The test 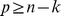 allows us to reject the rightmost *k*–lets motif because there is no right nucleotide it can be augmented with. The function *NucVal* maps a nucleotide to a numerical value (A, C, G, T→0, 1, 2, 3). It can be easily shown that no two different CanMotifs have the same encoding.

As we mentioned earlier, one of our concerns was avoiding of integer overflow while computing the encoding of CanMotifs. Our solution is to re-number the motifs sequentially starting from 0. The re-encoding scheme is straight forward (Algorithm 2). It goes over the entire encoded motif assigning a new code to each unique motif.

**Algorithm 2.** Algorithm to re-encode the *k*–lets motifs so it always starts from 0.

**Input.** List of encoded *k*–lets motifs 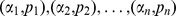 in increasing order, i.e. 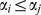 for all 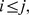 where the tuple stands for (encoded *k*–lets motif, its starting position)

**Output.** List of re-encoded *k*–lets motifs 
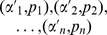


**Begin.**

*new_encoding* ← 0

*old_encoding* ← *α*_1_

**for** 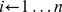 **do**

**{**

**if** (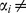 *old_encoding*)

**{**

*old_encoding* 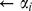


*new_encoding* ← *new_encoding*+1

**}**


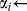
 *new_encoding*

**}**

**End.**

Back to our initial example *S* = TATAC, the encoded 2–lets motifs were {(12, 0), (12, 2)} where 12 is the encoding of 2–lets motif, TA (Table 1). The other number in the tuple, 0 and 2 in our case, represents the starting position of the motif. The algorithm returns the generated 3–lets encoded CanMotifs {(64, 0), (62, 2)}. For this computation, the algorithm sets the value of 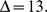 So we have two different 3–lets CanMotifs, one whose encoded value is 62 (CanMotif TAC), and the other is 64 (CanMotif TAT). Although we can determine what actual CanMotifs these are (using the starting position and the value of *k*), this is unnecessary for our task. The important thing is whether we have other CanMotifs of similar values?

### Sorting and discovering motifs

Given a list of encoded 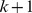
–lets CanMotifs the simplest way to discover the motifs is by sorting this list. It is a better method than doing an exhaustive search for each CanMotif. But the fastest sorting algorithm has a time complexity 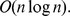 We, however, will use counting sort, a linear time sorting algorithm.

Using counting sort, we can sort the list of encoded 2–lets CanMotifs using 2 passes only. In the first pass, we count the number of occurrences of each distinct key value. That is, make a histogram of all encoded 2-lets AA, AC, AG, … etc., and in the second pass, we use arithmetic on this count to determine the position of each key value. As counting sort uses key values as indexes into the list, so it is linear in the size of the input list. Thus the cost of this algorithm is 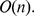


For 3–lets and beyond the situation is as follows. Given a sorted list of encoded *k*–lets motifs 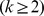 we generate a sorted list of encoded 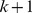
–lets CanMotifs. Initially we feed the encoded 2–lets motifs (sorted using counting sort) to get a sorted list of encoded 3–lets CanMotifs. For subsequent values of *k* the input is a subset of the output of the previous stage. So if the output is sorted that means the input to the next stage is sorted too. Algorithm 1 already generates the desired encoded CanMotifs; we only need to modify it to output in a sorted form.

**Figure 3:**
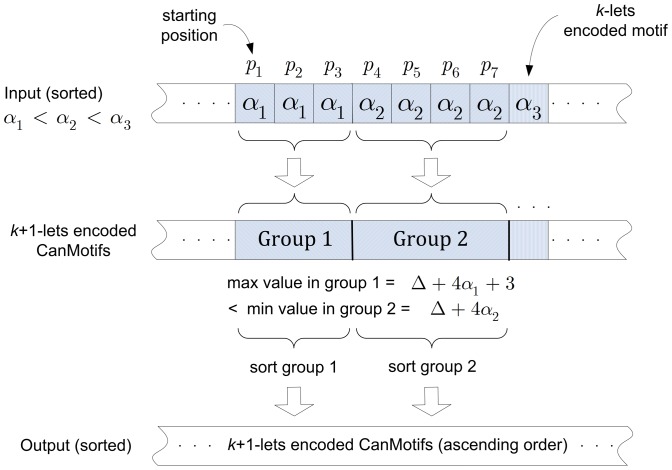
A linear algorithm to generate a sorted list of encoded 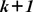
–lets CanMotifs from a sorted list of encoded *k*–lets motifs. Each group is sorted individually.

Suppose that 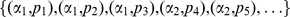 is the sorted list of encoded *k*–lets motifs, where 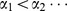 are the encoded motifs and 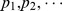 their starting positions. In generating the encoded 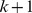
–lets CanMotifs, we note that the largest value 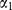 goes to is 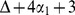 while the smallest value 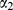 goes to is 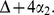 We can easily see that the relation 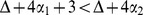 is true for any integer values of 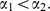 This suggests a naïve sorting algorithm where we do 4 passes (a pass for each nucleotide) through each group. All entries having 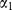 (as input) are a group, and those having 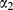 form another group and so on. Figure 3 illustrates the algorithm. The cost of sorting is,
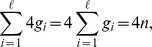
where 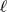 is the number of groups (different motifs) and 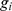 is the size of group 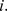 It is possible to slightly save on the cost of sorting if we use counting sort, however, complexity wise it will remain the same. So the cost to sort the generated list of encoded 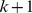
–lets CanMotifs is 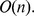


Now discovering the motifs is a trivial task. It is a matter of going through the sorted list of encoded 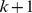
–lets CanMotifs, deleting any entry which occurs less than 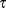 times. What is left is a sorted list of encoded 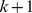
–lets motifs.

### The algorithm

The complete algorithm is shown as Algorithm 3. All the calculations are done in the array *B*, where at iteration 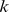 the *B[j].value* holds the encoding of the 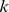
–lets which starts at position *B[j].pos*.

**Algorithm 3.** The full listing of the identical string motif discovery algorithm. The algorithm automatically keeps discovering larger and larger motifs and stops when there are no more motifs to be found.

**Input.** *Seq[ ].Nucleotide* the input sequence of size *SeqLength*, and threshold 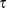


**Output.** Display the full list of identical string motifs

**Begin.**

**1** Initialize *Seq[ ].NucVal* with the corresponding nucleotide value (A, C, G, T→0, 1, 2, 3)

**2**

**3** Fill *Seq[ ].value* with the encoding of the pair bases (see Table 1), where *Seq[i].value* is the encoding for the pair at *Seq[i..i+1].Nucleotide*

**4**

**5** Count sort *Seq[ ]* on .*value* field in ascending order saving *B[j].value←Seq[i].value* and *B[j].pos←i*

**6**

**7** *k*←2

**8** *n←SeqLength – 1*

**9** **while** (*n*>0) **do**

**10** **{**

**11**  //remove all motifs that occur less than the threshold 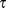


**12** 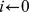


**13** **while** 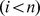 **do**

**14** **{**

**15**   Let 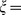
number of entries that has the same .*value* as *B[i].value*

**16** **if** 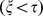


**17** **{**

**18**    Mark *B[i]*, *B[i+1]*, …, *B[* 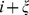 *]* for deletion

**19** **}**

**20** 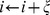


**21**  }

**22**  Discard all marked entries in array *B* by shifting the contents

**23**

**24** *c*←new size of the array *B* //number of occurrences of all *k*-lets motifs

**25**

**26** **for** *i* 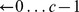 **do** renumber the different motifs starting from 0

**27** *Last*←1+largest motif number

**28** *n*←*c*

**29**  Output *k*-lets motifs and their starting positions

**30**

**31** *q* 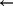
0

**32** *k* 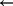 *k*+1

**33** **loop** over each group in array *B* //each group has the same .*value*

**34** **{**

**35** 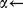
index of the first element in the group

**36** 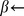
index of the last element in the group

**37** **for** *i* 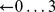 **do**

**38** **{**

**39** **for** 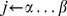 **do**

**40** **{**

**41** *x←B[j].pos*+*k*−1

**42** **if** (*x*<*SeqLength* && *Seq[x].NucVal =  = i*)

**43** **{**

**44** *tmp[q].value←Last*+4*B[j].value+*i*

**45** *tmp[q*++*].pos←B[j].pos*

**46** **}**

**47** **}**

**48** **}**

**49** **}**

**50** *B←tmp*

**51** **}**

**52** **End.**

The code should be simple to follow. The while-loop at lines 13–21 expect a list of all CanMotifs sorted on their encoding value. Since the list is sorted, we can determine the occurrence of each motif easily and remove those which occur fewer than the threshold 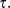 We re-encode (re-number) the encodings of motifs so that it always start from 0. The idea is to prevent an integer overflow, see the discussion in (Section CanMotifs encoding scheme.) This is achieved by the loop at line 26 (see Algorithm 2 for the details). Lines 31–49 code the algorithm in Figure 3, which generates a sorted list of encoded 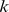
–lets CanMotifs from a sorted list of 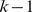
–lets motifs.

**Figure 4:**
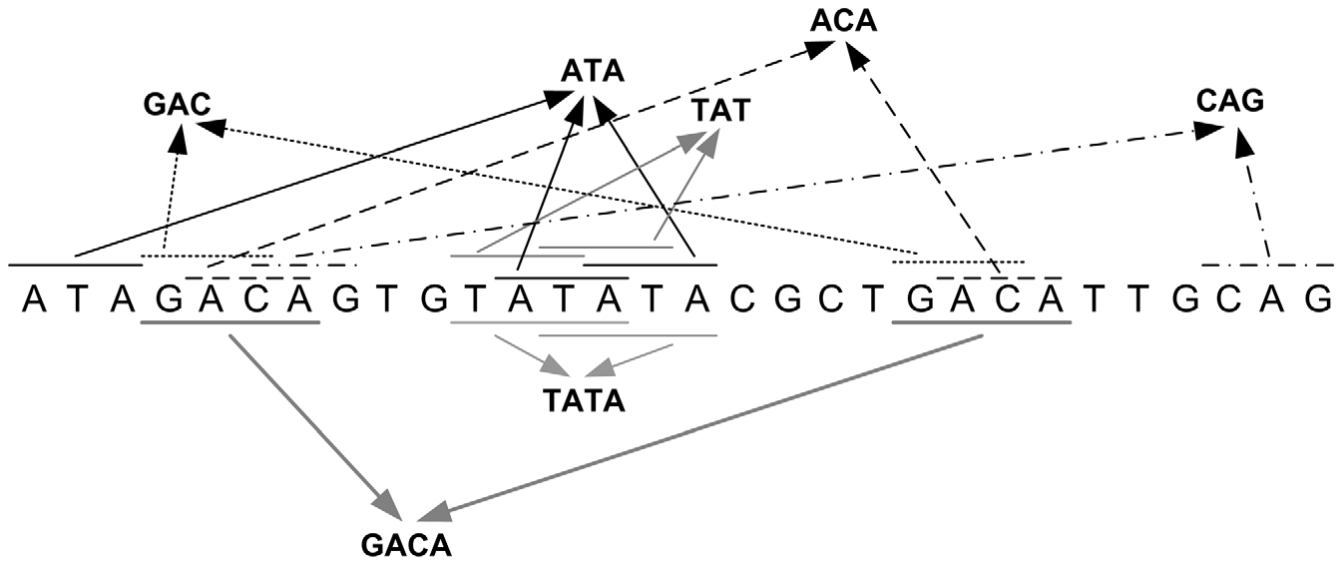
All the 3 and 4–lets identical string motifs in the sample sequence.

When applying the algorithm on the sample sequence below,
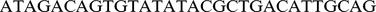
using the threshold 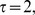 the algorithm discovers 9 different 2–lets motifs, 5 different 3–lets motifs and two different 4–lets motifs. Figure 4 marks all the 3–lets and 4–lets motifs in the example sequence.

The algorithm discovers and reports all motifs including those that overlap, e.g. TATA (Figure 4). Surely, overlapped motifs may not make sense biologically, but the algorithm does report them. These motifs can be easily discarded through a simple conditional statement that ensures the starting positions of any two 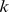
–lets motifs is at least 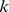 symbols apart.

### Complexity analysis of the algorithm

For the complexity analysis of the algorithm, we assume the size of the input sequence is 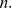 Starting with computational complexity. Line 1 requires 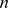 operations. We can use a hash table to initialize the *Seq[ ].value* with the encoding of pair bases (line 3) which costs 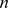 operations. In (Section Sorting and discovering motifs) we have shown that sorting 2–lets CanMotifs (line 5) costs 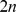 operations. The while-loop (lines 13–21) is used to mark all CanMotifs if they happen to occur less than the threshold 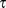 times. This can be efficiently implemented using 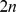 operations. In the first pass, we just mark those CanMotifs destined for removal with an invalid encoding, say −1, then deleting them (line 22) in the second pass by shifting the contents. A single pass is sufficient to re-number the motifs. The loop to generate the sorted list of encoded CanMotifs (lines 33–49) costs 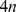 operations as shown in (Section Sorting and discovering motifs). The copying in line 50 costs 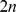 operations. Note that the main while-loop (lines 9–51) is repeated 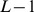 times, where 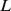 is the length of the longest motif that occurs not less than the threshold 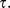 The theoretical worst-case time complexity is 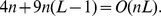 In reality, the time complexity is much lower. Note that 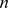 is the initial size of the input sequence, and this 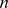 actually shrinks at each iteration since we are removing all CanMotifs that occur below the threshold.

For space complexity, we have three arrays all of size 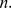 The array *Seq* has three components (*Nucleotide*, *NucVal* and *value*), while arrays *B* and *tmp* both have two components each (*pos* and *value*). The total space requirement is 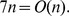


## Experimental Results and Discussion

For testing purposes, we will conduct experiments using three different data sets. A data set which is extracted from real data set from TRANSFAC [27]; randomly generated sequences of different sizes; and real biological sequences. The first data set is the same set that is used by Karci [26]. This data set is made up of two different sets: real, and synthetic. The real data set is from TRANSFAC; while the synthetic data set (generic and markov), is created out of extracted data from TRANSFAC using two different schemes to randomly place the binding site [28]. The full data set can be downloaded from the site, http://bio.cs.washington.edu/assessment/download.html. The Result of running the algorithm on the first data set is in Table 3. This table will help readers verify the accuracy of our algorithm and at the same time compare it with the results in [26].

**Table 3:** Experimental results of running our algorithm on selected sets from the data sets [27], [28] using 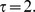

		No. string motifs(a)		
Sequences	k-lets	Overlapping		Examples with starting positions(b)
		No	Yes	
dm02r	11	2	15	ATCCCAATCCC→748, 760; ATCCCAATCCC→748, 760
	10	9	25	TTCTGCGGGC→670, 1164
	9	25	44	CTGCGGGCG→672, 1534
yst09r	23	2	2	GAAAAAAAAAAAAAAAAAAAAAA→11659, 13120
	17	3	3	
	16	5	5	TGAAAAAAAAAAAAAA→861, 11658
	15	13	13	GGTTTAAGCGTGAGG→324, 1319
hm20r	42	1	4	ACTCGGGAGGCTGAGGCAGGAGAATCACTTGAACCCGGGAGG→24338, 64502
	41	3	7	GAGACCAGCCTGGCCAACATGGTGAAACCCCGTCTCTACTA→4490, 58687
dm01g	14	2	2	CAGCGGCAGCAGCA→1372, 1393
				TGCCTATCGATAGT→3658, 5289
	13	5	6	TTATTATATATTT→32, 55
	12	11	13	
	11	28	32	
mus03g	12	2	2	TCTCCAAATCTA→755, 1103; CTCTTGGGAGCT→1575, 2400
	11	4	5	
	10	13	15	
yst01g	14	1	2	GATCTCAAAACAAA→4985, 6920
	13	3	5	GAACCAAAGATGG→506, 2239
	12	12	16	CTAAAAGAGTAA→2611, 4585
	11	37	44	ACCAAAGATGG→508, 2241
hm20m	18	1	1	TGCGCCAGGGCTGGGCTG→34498, 69949
	17	3	3	GCCCAGGGCTCCGCCGG→25468, 47769
	16	7	7	CCTGCAGCCCCCTCCC→5487, 69910
	15	17	19	AATGCTCCCCACGCC→35282, 59636
	14	47	52	GCCCTCAGCCGCGC→2385, 26115

(a) Count the number of different motifs. For non-overlapping motifs we only consider motifs if their starting position is further apart than their length.

(b) The starting position is based on index starting at 0. We followed [26] in treating each of the sequences as a single string. For example, yst09r.fasta is composed of 16 substrings each having 1000 nucleotides. These are merged into a single string with 16000 nucleotides.

This set includes real (sequences suffixed ‘r’), generic (sequences suffixed ‘g’), and markov (sequences suffixed ‘m’) data sets. Only larger sized identical string motifs are reported.

Next, we do a performance comparison to compare the execution time of our algorithm against the time needed by the algorithm in [26]. As stated earlier, this work is a significant improvement of the work in [26], which claimed a time complexity of 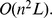 For the sake of fair comparison we implemented Karci's algorithm in MS Visual C#, which is the same environment that we used for our algorithm. Table 4 summarizes the execution time of both algorithms running on the same platform, an Intel core i5 processor based PC running at 2.67 GHz with 4 GB of RAM.

**Table 4:** Execution time (in seconds) to find identical string motifs of all sizes on an Intel core i5 based PC running at 2.67 GHz with 4 GB RAM.

Sequence	Size (# nucleotides)	Karci algorithm	Our algorithm
mus06r	1500	0.57	0.33
dm06r	3000	1.77	0.40
yst04r	7000	9.49	0.57
hm26r	9000	18.56	0.83
yst09r	16000	53.43	1.21
hm01r	36000	596.43	1.70
hm20r	70000	2225.15	2.45

**Figure 5:**
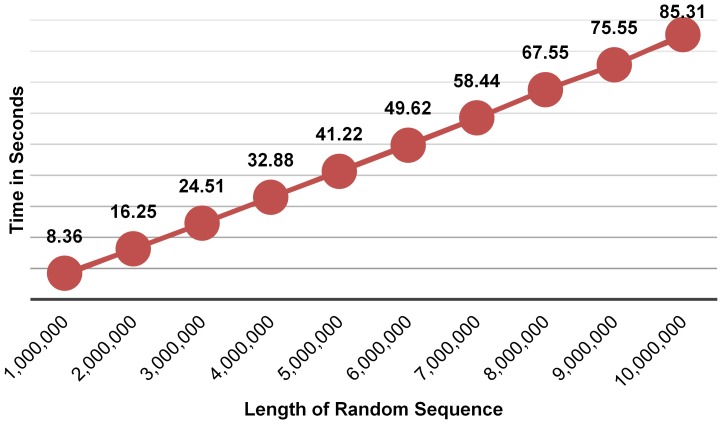
The average execution time (seconds) to discover all the identical string motifs of all sizes in 10 randomly generated sequences of each length. The algorithm clearly exhibits a linear behavior.

For further testing, we generated random sequences over the alphabet {A, C, G, T} of various lengths. For each length, we generated ten different random sequences and calculated the average time to discover all the identical string motifs of all possible sizes. Figure 5 plots the average time (in seconds) that our algorithm required for finding all the identical motifs. Using MS Excel, the time 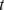 (in seconds) is given by 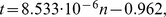 where 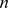 is the length of the sequence. The linear-in-time behavior of the algorithm is apparent.

The final test is on real biological sequences. Our algorithm was able to discover and report all the identical string motifs including some of impractical sizes. These huge motifs may not make sense biologically, but from a string point of view, they are valid identical motifs. For example, for Mesorhizobium opportunistum WSM2075 (NC_015675.1), a chromosome of length 6.88 million nucleotides, our algorithm found two identical string motifs of size 6357 nucleotides. Table 5 shows the time to discover all identical string motifs of lengths up to 40. We believe this is reasonable, since in reality motifs rarely exceed 40 nucleotides. Let 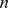 be the length of the sequence, then using MS Excel we can calculate the time (in seconds) which is 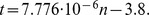


**Table 5:** Execution time (in seconds) to discover all the identical string motifs of lengths not exceeding 40 nucleotides in real biological sequences.

Organism	NCBI RefSeq	Size	Time
Vaccinia virus	NC_006998.1	0.19 M	1.83
Mycoplasma penetrans HF-2	NC_004432.1	1.36 M	10.38
Lactobacillus acidophilus NCFM	NC_006814.3	1.99 M	12.93
Methanocella paludicola SANAE	NC_013665.1	2.96 M	19.24
Acidiphilium multivorum AIU301	NC_015186.1	3.75 M	27.51
Mycobacterium tuberculosis H37Rv	NC_000962.2	4.41 M	28.81
Pectobacterium wasabiae WPP163	NC_013421.1	5.06 M	31.13
Mesorhizobium opportunistum WSM2075 chromosome	NC_015675.1	6.88 M	42.14
Saccharopolyspora erythraea NRRL 2338 chromosome	NC_009142.1	8.21 M	55.85
Caenorhabditis elegans Bristol N2 chromosome III	NC_003281.10	13.78 M	105.54
Caenorhabditis elegans Bristol N2 chromosome II	NC_003280.10	15.28 M	119.63

The size of the sequences is expressed in M (for millions).

Given that, the time function for finding all identical string motifs for random sequences as well as real biological sequences is close, this allows us to claim that our algorithm has indeed a complexity that is linear in time.

## Conclusion and Future Work

In this paper we presented an algorithm that discovers automatically all the identical string motifs in a given sequence. The idea of the algorithm is rooted in [26]; which had a complexity of 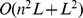 in time, and 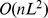 in space, where 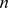 is the length of the input sequence and 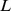 is the length of the longest possible motif. Our enhancement improved the complexity to 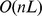 in time and linear in space. We were able to achieve this due to three factors: an encoding scheme for the motifs by which we have eliminated string comparison operation; relying on motifs only to generate a list of candidate motifs of larger size, which helps in placing a cap on the number of motifs to check among; and the usage of a linear algorithm to sort the encoded motifs thereby simplifying the task of identifying motifs. A further enhancement is the introduction of a threshold for the minimum number of occurrences of a motif. Experimental results on random, synthetic, and real biological sequences demonstrate that our algorithm has a time complexity that is linear.

For future work, we seek to enhance the algorithm so that it can discover the planted 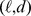 motifs, and the degenerate motifs.

## Implementation and Availability

The program is implemented in MS Visual C# running under Windows operating system. The executable is available for academic use only. It is obtainable through email request.
